# Changes in Upper Airway Airflow After Rapid Maxillary Expansion Beyond the Peak Period of Adenoidal Growth—A CBCT Study Using Computer Fluid Dynamics and Considering Adenoidal Dimensions as a Factor

**DOI:** 10.3390/dj13050209

**Published:** 2025-05-13

**Authors:** Giuseppe Palazzo, Rosalia Leonardi, Gaetano Isola, Manuel Lagravere, Antonino Lo Giudice

**Affiliations:** 1 Department of General Surgery and Medical-Surgical Specialties, Section of Orthodontics, University of Catania, Policlinico Universitario “Gaspare Rodolico—San Marco”, Via Santa Sofia 78, 95123 Catania, Italy; gpalazzo@unict.it (G.P.); rleonard@unict.it (R.L.); 2 Department of General Surgery and Medical-Surgical Specialties, Section of Periodontology, University of Catania, Policlinico Universitario “Gaspare Rodolico—San Marco”, Via Santa Sofia 78, 95123 Catania, Italy; gaetano.isola@unict.it; 3 Orthodontic Graduate Program, University of Alberta, Edmonton, AB T6G2R3, Canada; 4 Department of General Surgery and Medical-Surgical Specialties, Section of Pediatric Dentistry, University of Catania, Policlinico Universitario “Gaspare Rodolico—San Marco”, Via Santa Sofia 78, 95123 Catania, Italy

**Keywords:** maxillary expansion, upper airways, digital orthodontics, RME, 3D imaging, CBCT

## Abstract

Background/Objectives: This retrospective study used computer fluid dynamics (CFD) to evaluate the medium-term changes in the upper airways (UA) airflow after rapid maxillary expansion (RME) in three age-matched groups with different degrees of adenoidal obstruction. Methods: The sample included Cone-Beam Computed Tomography (CBCT) of 67 adolescents taken before (T0) and 12 months after RME (T1) and divided into three cohorts: Control Group (CG, <25% obstruction: 24 subjects, mean age = 11.8 ± 1.3), Adenoids Group 1 (AG1, >25% <75% obstruction: = 22 subjects, mean age = 10.9 ± 1.5), Adenoids Group 2 (AG2, >75% obstruction: = 21 subjects, mean age = 11.2 ± 1.6). The airflow pressure, velocity and obstruction were simulated using computer fluid dynamics (CFD). Results: The pressure significantly improved in CG and AG1 groups while the velocity improved in AG1 as well as the prevalence of obstruction improvement. The airflow pressure and velocity changes could be attributed to the reduction of the resistances in the adenotonsillar region, which was remarkably more marked in the AG1. Conclusions: Alterations in the adenotonsillar region likely represent the most substantial factors influencing airflow changes after RME. The integration of anatomical and functional data, along with the identification of baseline patient characteristics, may facilitate the characterization of phenotypes most appropriate for initial management through either Rapid Maxillary Expansion (RME) or otolaryngologic (ENT) interventions.

## 1. Introduction

Rapid Maxillary Expansion (RME) is widely recognized as the first-line orthopedic intervention for correcting transverse maxillary deficiency in growing individuals [1]. This technique involves the application of controlled forces through a maxillary expander—designed with tooth-borne, bone-borne, or hybrid anchorage—to induce the separation of the midpalatal suture [1,2,3]. Given that the maxilla forms the structural base of the nasal cavity, this orthopedic expansion results not only in transverse skeletal changes but also in a concomitant widening of the nasal airway [4,5]. Numerous studies have demonstrated that RME can significantly enlarge nasal cavity volume and lower airflow resistance, thereby improving nasal breathing efficiency [6,7,8].

Enhanced nasal patency following RME may contribute to improved airflow dynamics throughout the upper airway, potentially mitigating inspiratory effort and reducing the risk of pharyngeal airway collapse [9]. These functional improvements have led to the inclusion of RME in interdisciplinary strategies for managing pediatric obstructive sleep apnea (OSA). Nevertheless, current evidence supporting this application remains of low certainty, and its effectiveness varies considerably among patients [10]. Indeed, 50–60% of individuals undergoing RME do not report significant relief from nasal obstruction symptoms [11,12]. This variability may stem from individual anatomical, physiological, and pathological differences in the nasal and pharyngeal airway structures [13]. Of particular interest are the adeno-tonsillar tissues, whose size and inflammatory status appear to play a critical role in modulating airway resistance and, consequently, the functional outcomes of RME [13]. A recent study utilizing computational fluid dynamics (CFD) reported minimal airflow improvement in patients with concurrent nasal mucosal hypertrophy and adenoidal obstruction following RME [14]. That study included a wide age range (6–13 years), corresponding to a period of intense craniofacial development and high skeletal responsiveness to expansion. However, this developmental variability—especially the natural regression of adenoidal tissue beginning around age 7—may have introduced bias by overestimating treatment efficacy in younger children [15]. To address this limitation and better isolate the impact of adenoidal trophism on airway ventilation post-RME, it may be advantageous to investigate a more chronologically homogeneous population that extends beyond the peak of adenoidal hypertrophy. At this stage, growth-related changes are less likely to confound outcomes, as the lymphoid tissue typically undergoes involution and the immune system reaches greater maturity. For this reason, recent literature has encouraged studies focusing on narrow age brackets to better understand RME’s potential in improving upper airway airflow [16].

The present retrospective cohort study aimed to assess medium-term changes in airflow parameters—specifically, pressure and velocity—in the upper airway following RME, using CFD simulations based on CBCT data. The sample included three cohorts of subjects aged 11–14 years, each presenting with a different degree of adenoidal obstruction. Three-dimensional airway models were reconstructed from pre- and post-treatment CBCT scans. CFD, a validated computational technique derived from fluid mechanics, enabled the simulation of respiratory airflow through these anatomically accurate models, providing quantitative insights into intra-airway pressure and velocity during inhalation [17,18]. The study tested the null hypothesis that no significant intergroup differences in airflow changes would occur following RME.

## 2. Materials and Methods

### 2.1. Study Sample

This retrospective cohort investigation received ethical clearance from the Institutional Review Board of Indiana University–Purdue University (IRB protocol number: Pro00075765) and adhered to the ethical principles of the Declaration of Helsinki. The study enrolled growing patients diagnosed with maxillary transverse deficiency and managed with rapid maxillary expansion (RME) at the University of Alberta. Eligibility criteria included: (1) age between 11 and 15 years, (2) high-resolution CBCT images captured prior to expansion (T0) and 12 months following active treatment, before initiating fixed appliance therapy, (3) no previous orthodontic interventions, (4) no craniofacial abnormalities, (5) no history of adenoid or tonsil removal, and (6) absence of systemic illnesses or concurrent pharmacological therapy. All scans were performed using a standardized iCAT CBCT device (Imaging Sciences International, Hatfield, PA, USA). The acquisition settings involved an isotropic voxel size of 0.3 mm, an 8.9-s scan duration, a broad field of view, and parameters set to 120 kV and 20 mA. The spacing between slices was 0.3 mm, ensuring accurate anatomical correspondence. Participants were instructed to maintain a neutral head position, with the tongue relaxed and the tip touching the lingual surfaces of the lower incisors [19]. The imaging room featured adjustable lighting to optimize visualization during acquisition and preparation. RME was administered using a Hyrax-type expander with bands cemented on the first molars and extended arms reaching the permanent canines. Activation was performed once or twice daily until overexpansion was achieved, defined as the contact between the mesio-palatal cusps of the upper first molars and the buccal cusps of the opposing lower molars. The appliance remained in place for six months, serving as a passive retainer, during which no additional orthodontic appliances were employed.

The subjects were categorized into three groups based on the nasopharyngeal airway patency, measured on midsagittal CBCT sections as the distance from the posterior contour of the soft palate to the nearest adenoidal point: Control (Normal) Group (CG, <25% obstruction: 24 subjects, mean age = 12.8 ± 1.3), Adenoids Group 1 (AG1, >25% <75% obstruction: 22 subjects, mean age = 11.9 ± 1.5), and Adenoids Group 2 (AG2, >75% obstruction: 21 subjects, mean age = 12.2 ± 1.6) (Table 1).

### 2.2. CBCT Assessment

#### 2.2.1. Preliminary Definition of Mid-Palatal Suture (MPS) Maturation

Before conducting the segmentation and analyzing the simulated airflow dynamics, a preliminary evaluation was undertaken with two primary objectives: (1) to classify the maturation stage of the midpalatal suture (MPS) following the criteria outlined by Angelieri et al. [20], and (2) to detect any deviation of the nasal septum (NSD) through standardized diagnostic procedures [14,21]. Findings related to MPS development and NSD prevalence, along with any sex-based differences, are detailed in Table 1. All assessments were performed using Mimics software (v21.0; Materialise, Leuven, Belgium).

#### 2.2.2. Segmentation Protocol of Upper Airways (UAs)

**Figure 1:**
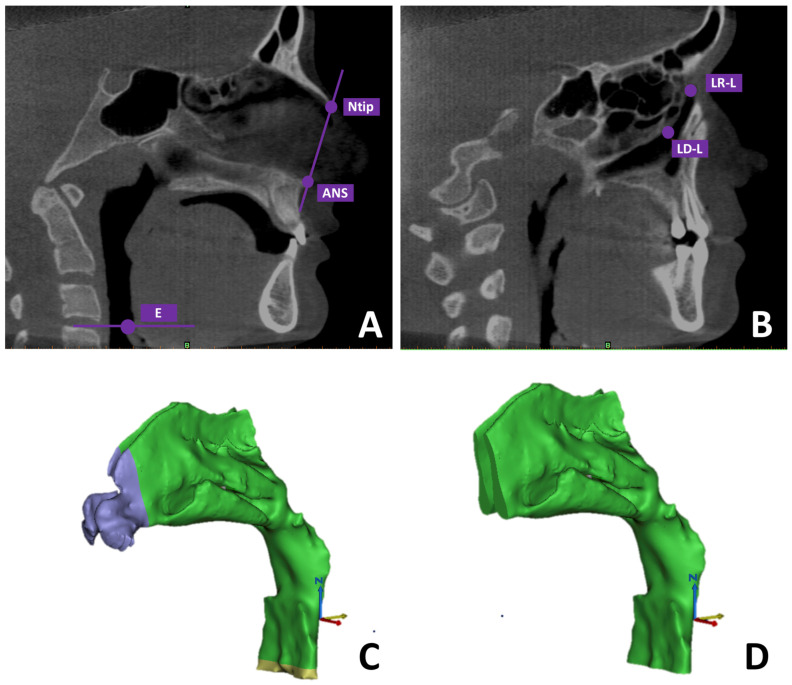
Determination of the anatomical limits used for the 3D evaluation of the upper airways: (**A**) the anterior border was defined by the plane connecting the nasal tip (Ntip) and the anterior nasal spine (ANS), while the posterior boundary was set by a plane passing through the epiglottis (E) and oriented parallel to the Frankfort horizontal plane; (**B**) the lateral margins were established by the location of the right and left lacrimal ducts, identified from the centroid of the lacrimal foramen to the lowest point of the right duct; (**C**) 3D reconstruction of the upper airway space illustrating the regions excluded from the analysis using sectional planes (purple zone = Ntip–ANS plane; yellow zone = epiglottis plane); (**D**) completed 3D airway model after refinement.

The same imaging software was utilized to carry out a semi-automatic segmentation of the upper airway (UA). Within the sagittal section, the anatomical landmarks were set as follows: the anterior boundary was defined by a plane connecting the nasal tip (Ntip) to the anterior nasal spine (ANS) (Figure 1A), while the lower limit was established using a plane through the epiglottis (E), oriented parallel to the Frankfort horizontal plane (FH) (Figure 1A). For lateral delimitation, the segmentation extended from the centroid of the lacrimal foramen to the lowest point of the right lacrimal duct, encompassing both sides (Figure 1B) [22,23]. Segmentation masks were created through an interactive thresholding process, where the operator selected the most appropriate intensity range to visualize the airway contours accurately. Subsequent refinement was performed manually on each axial slice to remove the paranasal sinuses—specifically the maxillary, frontal, ethmoid, and sphenoid sinuses—from the dataset. A final 3D reconstruction of the upper airway was then produced (Figure 1C,D).

#### 2.2.3. Skeletal Measurements

**Figure 2:**
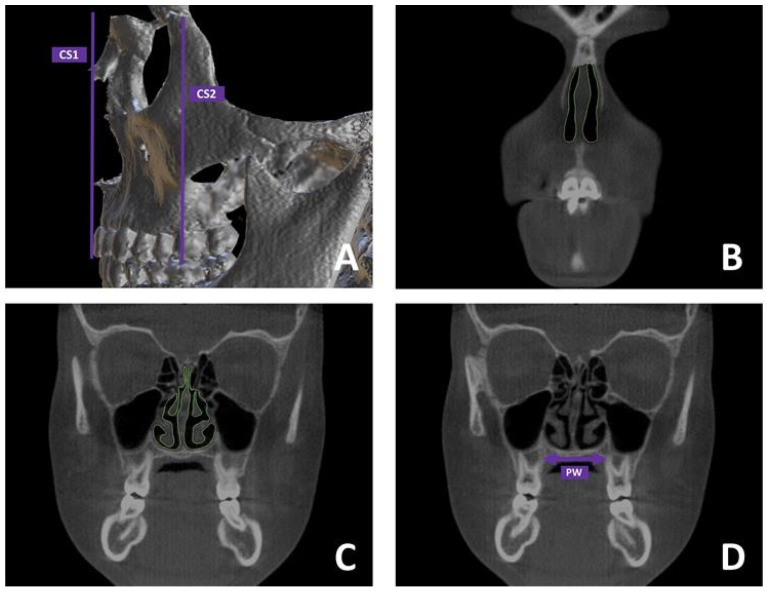
Skeletal assessments were conducted as part of this study. (**A**) Cross-sectional areas of the nasal cavity were defined and measured at two levels: one at the anterior nasal spine (CS1), and the other at the level of the maxillary first molars (CS2); (**B**) Coronal image illustrating the measurement site for CS1; (**C**) Coronal image illustrating the measurement site for CS2; (**D**) The transverse skeletal width (PW) was determined by measuring the distance between the points where the hard palate intersects with the lingual aspect of the alveolar bone.

The transverse dimension of the maxillary skeleton was determined on coronal sections by measuring the span between the interfaces of the hard palate and the lingual aspect of the alveolar bone (Figure 2A). This assessment was performed at the level corresponding to the furcation of the maxillary first molars.

In addition, cross-sectional areas (CSAs) of the nasal passages were analyzed on the same coronal slices, considering both the anterior and posterior regions of the airway (Figure 2B). The anterior cross-sectional area (CS1) referred to the area passing through the anterior nasal spine (Figure 2C), while the posterior cross-sectional area (CS2) referred to the area passing through the apex of the maxillary molar palatal root (Figure 2D).

#### 2.2.4. CFD Analysis

Prior to initiating computational fluid dynamics (CFD) analysis, the raw three-dimensional upper airway (UA) structures were processed using Rhinoceros (Version 7-Robert McNeel & Associates, TLM, Inc., Seattle, WA, USA), generating smooth surfaces while preserving the anatomical morphology. The resulting models were saved in STL (stereolithography) format and subsequently imported into ANSYS R2 CFD software (Version 24.2-ANSYS, Inc., Canonsburg, PA, USA) for simulation purposes. In accordance with prior CFD research on upper airway airflow, the airstream was treated as a Newtonian, incompressible, and homogeneous fluid [24]. The simulation framework employed elliptic-staggered governing equations along with the continuity principle [25]. The CFD was configured based on the following conditions: (1) air volume flowing at 200 mL/s, adjusted for the subjects’ growth stage, (2) no slippery wall surfaces, and (3) 1000 repetitions of the simulations to compute the mean values [14,17,21]. Each simulation was conducted twice, using two inlet configurations: one at the nostrils and the other at the oropharynx entrance. As no significant differences in airflow characteristics (pressure and velocity) were found between the two setups, the results from the nostrils during inspiration were used. Convergence was evaluated by tracking the residuals of mass and momentum, normalized to their respective inlet flux values. The process continued until all residuals fell below 0.2%. Maximum nasal airway pressure and velocity were measured to assess airflow dynamics. Obstruction was assumed when pressure exceeded −100 Pa, and velocity surpassed 10 m/s. This threshold matched a resistance value of 0.5 Pa/mL/s, reported in pediatric nasal obstruction studies [26], multiplied by the flow rate (200 mL/s). All tasks related to segmentation, skeletal measurements, and CFD analysis were performed by one operator (A.L.G.). After four weeks, the same operator repeated the measurements to assess intra-operator consistency. For inter-operator reliability, a second trained operator (G.P.) carried out the same process four weeks later.

### 2.3. Statistics

A preliminary power analysis was conducted on 20 subjects (10 in the CG group and 10 in the AG1 group). The analysis indicated that 21 participants per group were necessary to achieve 80% power for detecting a mean difference of 19.73 m/s in the inter-timing changes (T1–T0) between groups, with a 95% confidence level and a 20% beta error.

Descriptive statistics were calculated to evaluate the demographic and anatomical traits of the study sample, with a focus on identifying potential confounding factors. Comparisons of age and categorical variables (such as sex, skeletal maturity, and nasal septum deviation) across the three groups were performed using one-way ANOVA and the Chi-square test. Both tests confirmed that the distribution of these variables was consistent (Table 1).

An initial data assessment was carried out using the Shapiro-Wilk and Levene’s tests to assess normality and variance homogeneity. As the data were not normally distributed, non-parametric tests were employed. The Wilcoxon rank-sum test was used for intra-group comparisons of inter-stage differences, while Kruskal-Wallis and Mann-Whitney U tests were applied to compare baseline median values and median inter-stage differences (T0-T1) between the groups. The McNemar and Chi-square tests were used for comparing categorical variables (e.g., obstruction) across treatment stages and between groups. Spearman’s rank correlation coefficients (rs) were calculated to assess correlations between CFD parameters (pressure and velocity) and skeletal parameters (CS1, CS2, PW). Intra-examiner reliability was evaluated with the Intraclass Correlation Coefficient (ICC), and the method error was calculated using Dahlberg’s formula. Statistical significance was set at *p* < 0.05. Data were analyzed using SPSS^®^ version 24 (IBM Corporation, 1 New Orchard Road, Armonk, New York, NY, USA).

## 3. Results

### 3.1. Reliability Assessment

The ICC values recorded ranged from 0.864 to 0.922 for intra-operator variability and from 0.841 to 0.876 for inter-operator variability, suggesting the entire digital build-up was highly reliable. According to Dahlberg’s formula, the random error for skeletal measurements was 0.11 mm (PW), 0.397 mm^2^ (CS1), and 0.509 mm^2^ (CS2), and the random error detected for CFD data was 1.11 Pa (pressure) and 0.285 m/s (velocity).

### 3.2. Baseline Data

The median values for functional parameters (pressure and velocity) were notably higher in the AG1 and AG2 groups compared to the CG, indicating greater resistance in the former. However, no significant differences were found in the median values of skeletal parameters across the three groups. This suggests that craniofacial development is similar among all groups, as evidenced by the consistent distribution of participants in each group according to their skeletal maturation stage.

### 3.3. Inter-Timing Changes (T0–T1)

**Figure 3:**
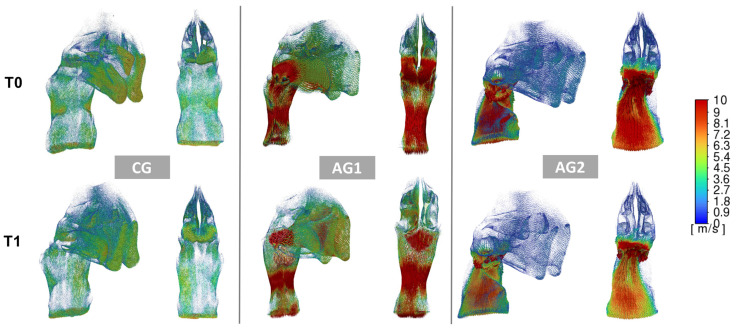
Plots illustrating the airflow velocity pattern at baseline (T0) and 12 months following RME (T1). The left panel illustrates an individual from the CG, the middle panel represents an individual from AG1, and the right panel shows an individual from AG2. Within each panel, a 3/4 view of the upper airways (UA) is presented on the left, alongside a posterior view of the UAs on the right. The airflow velocity was found to be elevated in the adenotonsillar region. At baseline, the airflow velocity was higher in both AG1 and AG2; however, post-treatment alterations were particularly pronounced in AG1.

**Figure 4:**
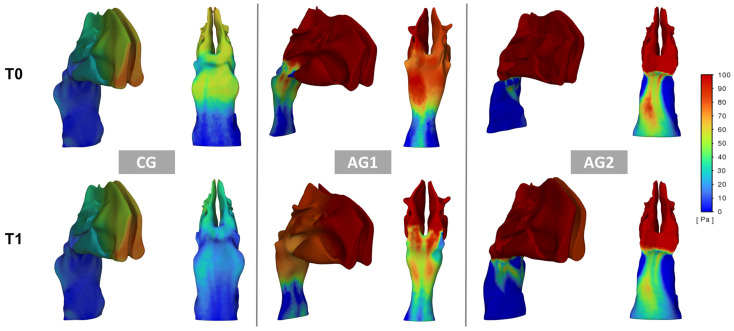
Plots illustrating airflow pressure patterns at baseline (T0) and 12 months after RME (T1). The **left** panel shows an individual from the CG, the **middle** panel represents an individual from AG1, and the **right** panel depicts an individual from AG2. Within each panel, a 3/4 view of the upper airways (**left**) and a posterior view (**right**) are displayed. Maximum pressure was generally observed in the anterior region of the nasal cavity, reflecting resistance within the pharyngeal region (with the inlet defined at the nostrils). At baseline, pressure values were higher in both AG1 and AG2. Following RME, a noticeable pressure reduction was observed in AG1.

Concerning functional parameters, a significant reduction of median pressure was found in NG and AG1 groups (change percentage = 14.53% (CG) and 19.68% (AG1), while a significant velocity of the airflow was found only in the AG1 (change percentage = 18.40%) (Figure 3 and Figure 4, Table 2). For skeletal measurements, the three groups showed a significant increment of PW (change percentage = 11.22% (CG), 10.38% (AG1), 9.97% (AG2)), CSA1 (change percentage = 20.01% (CG), 23.25% (AG1), 15.37% (AG2)) and CSA2 (change percentage = 15.08% (CG), 15.05% (AG1), 8.71% (AG2)) (Table 2).

### 3.4. Comparison of the Changes Among CG, AG1 and AG2 Groups

Regarding functional parameters, significant differences were observed among the three groups regarding the changes in pressure and airflow velocity, with the highest values recorded in the AG1 group, as confirmed by the post-hoc analysis. No significant differences were found among the groups in skeletal measurements at the PW and CSA1 levels. However, the AG2 group showed significantly smaller changes in CSA2 compared to both the CG and AG1 groups, according to the post-hoc analysis (Table 2).

Notable differences were also found in the baseline distribution of obstructive conditions across the groups, with a higher proportion of participants in the AG1 and AG2 groups exhibiting obstruction. However, CFD analysis revealed that the CG group had a significantly higher proportion of individuals with resolution of the obstructive condition at T1. Specifically, 7 out of 24 individuals in the CG group presented with obstruction at baseline and 6 of these 7 individuals showed resolution after RME. In comparison, 16 out of 22 individuals in the AG1 group had obstruction at baseline, with 8 of these 16 individuals showing resolution post-RME. Similarly, 16 out of 21 individuals in the AG2 group had obstruction at baseline, and only 2 out of 16 individuals showed resolution after RME.

### 3.5. Correlation Between Ventilation Parameters and Skeletal Parameters

No statistically significant correlations were found between pressure or velocity at the upper airways (UAs) and PW or CS1, either before treatment, after treatment, or in the inter-timing differences (T0-T1) across the three groups. A weak negative correlation was observed between pressure, velocity, and CS2 at baseline in all groups, as well as in the inter-timing differences (T0-T1) specifically in the AG1 group (Table 3).

## 4. Discussion

RME has been reported to improve nasal breathing based on a one-year follow-up [8]. However, given the lack of robust long-term data, caution should be exercised when considering RME as a preventive approach for enhancing respiratory function in growing children. For this reason, a recent consensus statement from the American Association of Orthodontists emphasized that the main purpose of RME is to correct maxillary transverse deficiency and improve occlusion, rather than to act as a respiratory treatment [27]. This caution is driven by the limited availability of well-conducted studies, the age diversity within study populations, and the variability in study settings [12,27]. Additionally, individual variations are often not adequately addressed when assessing the impact of RME on nasal airway obstruction.

### 4.1. Concerning

In terms of age-related growth changes, previous studies have indicated that adenotonsillar hypertrophy tends to regress naturally in children between 7 and 8 years of age, followed by spontaneous improvement [28]. It is well-known that the adenoids (and the Waldeyer’s ring) grow until around 5–7 years, after which they gradually shrink [15,28]. This period also coincides with the highest incidence of adenotonsillar hypertrophy in children of primary school age [29,30,31]. However, many studies investigating the impact of RME on respiratory function have included a wide age range, typically spanning from 6 to 13 years, which is considered the optimal period for achieving skeletal expansion of the midpalatal suture [20]. As a consequence, younger individuals may have experienced a reduction in lymphatic tissue hypertrophy compared to older individuals and a spontaneous improvement of upper airway obstruction that might be erroneously attributed to the success of RME therapy and not to normal craniofacial growth [32,33]. This was confirmed by a recent study where the authors found a significantly greater expansion of the rhinopharynx in the early treatment group (6–9 years old) compared to the late treatment group (11–14 years old), one year after RME [22]. In this context, it could be noteworthy to assess the airflow changes after RME in individuals with varying degrees of adenoidal obstruction and across an age range extending beyond the peak period of adenoidal growth. This information would provide new insights by analyzing the airflow changes after RME across a period in which growth-related changes due to adenoid maturation are not anticipated. To the best of our knowledge, this is the first study in the literature addressing this purpose.

We employed CFD simulations to examine the changes in upper airway airflow following RME, as this technology allows for an accurate evaluation of upper airway function by modeling air pressure and velocity. This approach provides a more comprehensive assessment compared to purely morphological analyses. [17] Previous CFD studies have primarily concentrated on post-RME airflow changes in the nasal cavity alone, without considering the adenoids, palatine tonsils, and soft palate. In contrast, our study included simulations of the entire oro- and nasopharynx since this approach allowed us to examine the airflow changes through the entire upper airways. Also, it allowed for a more accurate visualization of these results in relation to soft tissue morphological changes occurring due to growth, as illustrated in graphical plots (Figure 3 and Figure 4).

The effectiveness of skeletal expansion and upper airway (UA) volumes can be affected by different expansion protocols and the maturation stages of the midpalatal suture (MPS), which may also influence CFD measurements [34]. In this study, all groups utilized the same appliance design, specifically a Hyrax expander with bands on the first molars, and followed identical daily activation protocols. Additionally, all participants were at palatal maturation stages A or B, with no differences in distribution between the groups. This ensured sample homogeneity and accounted for the similar amounts of skeletal expansion observed in both groups, as indicated by the PW, CS1, and CS2 parameters.

It has been reported that the measurement of the upper airway (UA) dimensions can vary based on specific CBCT settings, including head posture [35], tongue posture [19], different scanning systems [36], and post-processing factors such as segmentation protocols and software. In this study, all radiographic examinations were conducted using the same CBCT machine and settings for both pre-treatment and post-treatment images, ensuring standardized tongue and head positions. The voxel size used (0.3 mm) was sufficient for accurately defining and reconstructing the UA anatomy in three dimensions [36].

The three groups exhibited significantly different values in air pressure and velocity at the baseline (Table 2). This finding could be attributed to the different trophic conditions of the adenoidal tissue in the three groups and is in agreement with previous evidence [18,37]. The plots in Figure 3 and Figure 4 clearly show the pattern of pressure and airflow in the three groups at baseline; in particular, the airflow pressure and velocity increased due to higher resistance in the nasopharyngeal region in groups AG1 and AG2, with a drastic increase in the upstream direction (nasal region) in AG2. It has been reported that the skeletal dimensions of the nasal cavities can affect the presence of nasal airway obstructions and that a cross-sectional area (CSA) of 250 mm^2^ can be considered a critical threshold below which the incidence of obstructions increases significantly [14]. However, in the present study, CSA1 and CSA2 values were consistently below the critical threshold and were similar across all three groups. This finding would confirm that, within certain dimensional skeletal limits (not yet defined), the physio-pathological conditions, dimensions, and morphology of the soft tissues are crucial in the formation of obstructive sites. Although all groups showed a reduction of airflow velocity and pressure one year after RME (T1–T0 median pressure: CG = −11.64 Pa, AG1 = −39.02 Pa; AG2 = −23.16 Pa; T1-T0 median velocity: CG = −0.72 m/s, AG1= −2.86 m/s, AG2= −1.95 m/s), the post-treatment changes were remarkably greater in the AG1, even when calculated as percentage values of the baseline data. Given that the three groups were homogeneous in terms of age and maxillary expansion protocol, this difference could be explained in relation to the different conditions of the adenoidal tissues. In this regard, two reasons could explain the greater positive responsiveness of the airflow found one year after RME in AG1. Firstly, it is plausible that subjects in this group may have experienced a residual regression of adenoid tissue (a growth-related phenomenon), which, in combination with an increase in pharyngeal diameters (also related to growth), could have facilitated improved airflow. Figure 3 demonstrates that the main inter-timing variations occurred in the rhinopharyngeal area, where post-treatment changes in airflow velocity were most prominent. Additionally, the reduction in adenoids could be linked to the alleviation of irritative conditions, possibly due to the decreased pressure and reduced collapsibility of the pharyngeal space following the lower resistance induced by RME—though this remains a hypothesis with limited supporting evidence. At the same time, the expansion of the posterior maxillary area might promote a corresponding adaptation or displacement of the surrounding soft tissues in the nasopharynx, thereby increasing the volume in this region [38]. This assumption may help explain the observed moderate negative correlation between the expansion of CS2 and improved ventilation parameters in the AG1 group. Different factors may explain why both the CG and AG2 groups exhibited smaller changes in pressure and velocity compared to AG1. In the CG, the existing patency of the pharyngeal airways limited the potential for airflow changes after RME, even when considering the positive impact of maxillary skeletal expansion on nasal resistance [5]. Conversely, in AG2, airflow likely did not improve due to the persistence of scarce patency pharyngeal conditions in the post-treatment stage. These results are consistent with previous otolaryngology studies and align with recent orthodontic research, although the inter-timing difference of airflow found in the CG is remarkably lower than those reported by Sakoda et al. [14]. A possible explanation for this discrepancy is that the latter study included participants with a wide age range (6–13 years) where growth changes in younger children could have overestimated the effectiveness of RME on upper airway ventilation.

Regarding skeletal changes, no statistically significant differences were observed among the three groups in the increases of PW, CS1, and CS2. These results support the idea that the changes in airflow dynamics were mainly driven by morphological and volumetric alterations in the surrounding soft tissues rather than the skeletal structures themselves. The expansion of CS1 and CS2 is partly attributed to normal craniofacial growth. A previous study reported an increase of approximately 12 mm^2^ for CS1 and 25 mm^2^ for CS2 over a 27-month period following RME [21]. When annualized (around 6 mm^2^ and 12 mm^2^, respectively), the dimensional changes observed in this study fall within the ranges reported in the existing literature (CS1: 20–25 mm^2^; CS2: 20–35 mm^2^) [14,18]. Moreover, the complex anatomy of the posterior nasal airway, along with growth-related changes in the pharyngeal soft tissues and individual variations, likely explains the absence of a strong correlation between cross-sectional area and upper airway resistance or velocity. The CFD analysis indicated the presence of obstructive conditions in some subjects both before and after RME. Functional obstruction was defined by pressure values exceeding 100 Pa at an inflow rate of 200 mL/s, a threshold consistent with previous studies and corresponding to an airway resistance above 5.0 cm H_2_O/L·s [37,39]. This level is close to the upper limit of normal nasal airway resistance reported in the otolaryngology literature for individuals around the mean age of the current study sample (approximately 12 years). According to this criterion, a significantly higher number of obstructed cases was found in the AG1 and AG2 groups, which can be explained by more pronounced lymphoid tissue hypertrophy at baseline compared to the CG group. Regarding improvement rates, the CG group showed the highest success rate, with half of the subjects in the AG1 group who had obstruction at baseline showing improvement. These improvement ratios align with previous studies suggesting that obstructions may resolve after RME, particularly when hypertrophic adenoids are present with an obstruction of less than 75% [14]. It is interesting to note from the plots that the (few) cases in the CG group with baseline obstruction exhibited resistances localized in the nasal region, which improved 12 months after maxillary expansion. This localized resistance was not observed in the other two groups, likely because, even if present, it would be secondary to the resistances in the nasopharynx, which are primarily responsible for the increased pressure and airflow throughout the upper respiratory tract, including the nasal region. This assumption is supported by observations in some individuals in the AG1 group, where the CFD identified areas of obstruction in the nasal region that were not detectable at T1, coinciding with improvements in obstruction at T1.

From a clinical standpoint, our findings underscore several critical considerations for the multidisciplinary management of patients exhibiting oral breathing habits, particularly emphasizing the role of orthodontists and the relevant guidelines from otolaryngology literature. In adolescents, such as those studied, various pathophysiological conditions affecting the pharyngeal soft tissues can influence upper airway ventilation following RME. For patients without nasopharyngeal resistance, substantial improvements in airflow post-RME are unlikely unless there is nasal resistance that may be addressed through skeletal maxillary expansion. In patients with nasopharyngeal obstruction <75%, it might be possible to observe an improvement in obstruction, airflow velocity, and pressure after RME, though the specific effects of RME (pharyngeal expansion support, reduced irritative stimulation of lymphatic tissue and mucosa due to improved nasal airflow) remains unclear. For these individuals, it might be advisable to combine maxillary expansion (only for those with a diagnosed cross-bite or transverse maxillary deficiency) with either topical or systemic pharmacological treatments aimed at alleviating the inflammatory condition of the upper airways, or with additional therapies such as myofunctional therapy. Conversely, patients with nasopharyngeal obstruction greater than 75% are unlikely to benefit from RME and should be considered for surgical intervention. Integrating anatomical and functional data, along with identifying baseline patient characteristics, could help in defining phenotypes best suited for initial treatment with either Rapid Maxillary Expansion (RME) or otolaryngologic (ENT) interventions. This integration is crucial for enhancing interdisciplinary treatment planning and clinical decision-making between otolaryngologists and orthodontic specialists. Future studies are needed to further explore and refine these collaborative approaches.

### 4.2. Limitations

- The primary limitation of this study is the absence of an untreated control group, which would have provided insights into the natural developmental changes of the upper airways in growing individuals. However, the use of CBCT scans in untreated controls raises ethical concerns due to unnecessary exposure to ionizing radiation in pediatric subjects [40,41]. While the control group in this study cannot fully replace an untreated control group, it did allow for the differentiation between two distinct pharyngeal tissue conditions: normal (adenoidal obstruction < 25%) and hypertrophic (AG1 and AG2).
- The results of this study cannot be generalized for several reasons: (1) the study focused on adenoidal hypertrophy as the primary factor, excluding other potential causes of obstruction (e.g., palatine tonsils, nasal valve collapse, soft tissue changes, etc.); (2) the sample was a retrospective orthodontic cohort that did not include otolaryngological examination. Future studies are encouraged to evaluate the effects of RME on upper airway ventilation with and without otolaryngological intervention, and (3) the anterior portion of the nasal cavity was excluded from the segmentation and CFD analysis due to low accuracy in 3D modeling of this region. Although including this area could have affected CFD patterns, such differences are likely of minimal comparative significance, as the main differences between the groups were linked to the patency of the posterior airways.

## 5. Conclusions

- Twelve months after treatment (T1), there was a slight improvement in the ventilation conditions (pressure, velocity, and obstructions) in both CG, AG1, and AG2. However, such increment was significantly greater in AG1 compared to the other two groups.
- The alterations in the adenotonsillar region likely represent the most substantial factors influencing UA airflow changes.
- A general weak tendency toward inverse correlation was found between the increment of CS2 and the improvement of ventilation parameters only in AG1.

Accordingly, twelve months after treatment, clinicians should not expect changes in the UAs dimensions to be solely related to the treatment effects of RME. In individuals with mild to moderate adenoidal obstruction (<75%), RME may aid in reducing obstruction, though the precise mechanisms remain unclear.

## Figures and Tables

**Table 1:** Demography and clinical characteristics of the sample of the study.

Sample	Total (n = 67)	Control Group (CG; n = 24)	Adenoids Group 1 (AG1; n = 22)	Adenoids Group 2 (AG2; n = 21)	Significance *	
Characteristics						
	Mean/n	Mean/n	Mean/n			
Mean age		11.8 (± 1.3)	10.9 (± 1.5)	11.2 (± 1.6)	NS	
Gender					NS	
*Male*		10	9	9		
*Female*		14	13	12		
MPS Stage					NS	
*Stage A*		11	14	10		
*Stage B*		13	8	11		
NSD						
*Yes*		6	7	8	NS	
*No*		18	15	13		

* *p*-value for comparison of group means by one-way analysis of the variance (ANOVA) or differences in proportion calculated by chi-square test.

**Table 2:** Inter-timing and inter-group differences for ventilation and skeletal parameters investigated in both Control/Normal (CG), Adenoids 1 (AG1) and Adenoids 2 (AG2) groups.

	CG (n = 24) (a)				AG 1 (n =22) (b)				AG 2 (n = 21) (c)			
	Median	IQR	*p* Value *		Median	IQR	*p* Value *		Median	IQR	*p* Value *	*p* Value **
*Airway Pressure* (Pa)												
T0	82.625	(54.25–102.43)	<0.05		198.24	(157.57–240.98)	<0.05		254.6	(236.73–297.10)	0.066	<0.05 (a-b, a-c)
T1	70.62	(42.53–88.56)			159.22	(127.90–181.25)			231.44	(187.11–264.46)		<0.05 (a-b, a-c, b-c)
T1-T2	12.01	(−3.74–22.21)	-		39.02	(4.69–60.16)	-		23.16	(8.59–66.09)	-	<0.05 (b-a, b-c)
Change (%)	14.53				19.68				9.10			
*Airway Velocity* (m/s)												
T0	6.65	(5.54–8.31)	0.092		15.54	(9.07–21.22)	<0.05		27.42	(16.51–31.06)	0.074	<0.05 (a-b, a-c, b-c)
T1	5.93	(4.11–7.43)			12.68	(6.39–15.92)			25.47	(22.55–35.75)		<0.05 (a-b, a-c, b-c)
T1-T2	0.72	(0.51–3.15)	-		2.86	(1.06–7.47)	-		1.95	(−6.06–6.96)	-	<0.05 (b-a, b-c)
Change (%)	10.83				18.40				7.11			
*PW* (mm)												
T0	24.16	(22.64–29.13)	<0.001		22.64	(20.89–25.19)	<0.001		23.68	(21.37–25.98)	<0.05	0.231
T1	26.87	(23.95–31.59)			24.99	(22.81–27.48)			26.04	(24.53–28.14)		0.379
T1-T2	2.71	(0.77–3.34)	-		2.35	(0.97–2.91)	-		2.36	(0.61–4.46)	-	0.406
Change (%)	11.22				10.38				9.97			
*CS1* (mm^2^)												
T0	166.09	(140.07–191.28)	<0.001		156.40	(126.28–175.10)	<0.001		198.11	(169.18–213.41)	<0.001	0.092
T1	199.32	(176.64–218.49)			192.76	(178.26–204.32)			228.56	(189.96–248.93)		0.121
T1-T2	33.23	(11.15–54.03)	-		36.36	(15.01–50.50)	-		30.45	(18.02–45.11)	-	0.532
Change (%)	20.01				23.25				15.37			
*CS2* (mm^2^)												
T0	235.4	(222.65–271.08)	<0.001		198.67	(190.01–235.91)	<0.05		217.25	(195.25–228.99)	<0.05	0.256
T1	270.89	(236.83–312.50)			228.56	(209.10–251.27)			236.17	(202.53–261.31)		0.441
T1-T2	35.49	(9.10–46.66)	-		29.89	(6.40–44.13)	-		18.92	(0.75–32.32)	-	0.358
Change (%)	15.08				15.05				8.71			
												
*Obstruction (n)*	*T0*	*T1*	***p*** value ***		*T0*	*T1*	***p*** value ****		*T0*	*T1*	***p*** value ***	***p*** value ****
YES	7	1	<0.001		16	8	0.151		16	14	0.606	<0.05
NO	17	23			6	16			5	7		

* *p*-value set at *p* < 0.05 and based on Wilcoxon rank-sum test (inter-stage differences). ** *p*-value set at *p* < 0.05 and based on Kruskal-Wallis test (inter-group change differences) and Mann-Whitney t-test for single comparisons. *** *p*-value set at *p* < 0.05 and based on McNemar test (intra-group distribution). **** *p*-value set at *p* < 0.05 and based on Chi-Square test (baseline inter-group distribution comparison). IQR = Interquartile Range; Pa = Pascal.

**Table 3:** Correlation coefficients (rs) based on Spearman rank test and *p* values of the relationship between ventilation parameters and skeletal parameters.

Groups	Timing	Airway Ventilation	PW				CS1				CS2		
	
			T0	T1	T1-T0		T0	T1	T1-T0		T0	T1	T1-T0
CG	T0	Pressure	−0.103	-			−0.074	-			−0.484 *	-	
		Velocity	−0.062				−0.265				−0.230		
	T1	Pressure	-	−0.194	-		-	−0.264	-		-	−0.571 *	-
		Velocity		−0.065				−0.347				−0.331	
	T1-T0	Pressure	-		−0.327		-		−0.067		-		−0.021
		Velocity			−0.25				−0.15				−0.072
													
AG1	T0	Pressure	−0.169	-			−0.227	-			−0.408	-	
		Velocity	0.033				0.109				−0.495 *		
	T1	Pressure	-	0.104	-		-	−0.234	-		-	−0.403 *	-
		Velocity		−0.204				−0.277				−0.580 *	
	T1-T0	Pressure	-		−0.283		-		−0.146		-		−0.415 *
		Velocity			0.114				−0.118				−0.498 *
													
AG2	T0	Pressure	−0.025				−0.125				−0.361		
		Velocity	−0.274				0.026				−0.302		
	T1	Pressure		−0.028				−0.235				−0.391	
		Velocity		−0.121				−0.341				−0.317	
	T1-T0	Pressure			−0.027				−0.131				−0.204
		Velocity			−0.26				0.164				−0.378

CG = Control (Normal) Group; AG1 = Adenoid Group 1; AG2 = Adenoid Group 2; CS1 = Anterior Cross-sectional Area; CS2 = Posterior Cross-sectional Area. * Statistical significance set at *p* < 0.05.

## Data Availability

Data are available upon request to the corresponding author.

## Abbreviations

UA	upper airways
RME	rapid maxillary expansion
CBCT	Cone-Beam Computed Tomography
CFD	computational fluid dynamics
MPS	maturation stage of the mid-palatal suture
NSD	nasal septum deviation
FH	Frankfort plane
ICC	Intraclass Correlation Coefficient
ENT	Ear, Nose, and Throat
